# BMVC test, an improved fluorescence assay for detection of malignant pleural effusions

**DOI:** 10.1002/cam4.179

**Published:** 2014-01-10

**Authors:** I-Ting Lin, Yu-Lin Tsai, Chi-Chih Kang, Wei-Chun Huang, Chiung-Lin Wang, Mei-Ying Lin, Pei-Jen Lou, Jin-Yuan Shih, Hao-Chien Wang, Huey-Dong Wu, Tzu-Hsiu Tsai, I-Shiow Jan, Ta-Chau Chang

**Affiliations:** 1 Institute of Atomic and Molecular Sciences, Academia SinicaTaipei, 106, Taiwan; 2 Taiwan International Graduate Program and Department of Chemistry, National Tsing-Hua University and Academia SinicaTaipei, Taiwan; 3 Department of Otolaryngology, National Taiwan University Hospital and National Taiwan University College of MedicineTaipei, Taiwan; 4 Department of Internal Medicine, National Taiwan University Hospital Yun-Lin BranchYun-Lin, Taiwan; 5 Department of Internal Medicine, National Taiwan University Hospital and National Taiwan University College of MedicineTaipei, Taiwan; 6 Department of Integrated Diagnostics and Therapeutics, National Taiwan University Hospital and National Taiwan University College of MedicineTaipei, Taiwan; 7 Department of Laboratory Medicine, National Taiwan University Hospital and National Taiwan University College of MedicineTaipei, Taiwan

**Keywords:** Cell-collecting, fluorescence probe, malignant pleural effusions

## Abstract

The diagnosis of malignant pleural effusions is an important issue in the management of malignancy patients. Generally, cytologic examination is a routine diagnostic technique. However, morphological interpretation of cytology is sometimes inconclusive. Here an ancillary method named BMVC test is developed for rapid detection of malignant pleural effusion to improve the diagnostic accuracy at low cost. A simple assay kit is designed to collect living cells from clinical pleural effusion and a fluorescence probe, 3,6-Bis(1-methyl-4-vinylpyridinium) carbazole diiodide (BMVC), is used to illuminate malignant cells. The fluorescence intensity is quantitatively analyzed by ImageJ program. This method yields digital numbers for the test results without any grey zone or ambiguities in the current cytology tests due to intra-observer and inter-observer variability. Comparing with results from double-blind cytologic examination, this simple test gives a good discrimination between malignant and benign specimens with sensitivity of 89.4% (42/47) and specificity of 93.3% (56/60) for diagnosis of malignant pleural effusion. BMVC test provides accurate results in a short time period, and the digital output could assist cytologic examination to become more objective and clear-cut. This is a convenient ancillary tool for detection of malignant pleural effusions.

## Introduction

An accumulation of fluid in the pleural cavity is known as a pleural effusion 1. The diagnosis of malignant pleural effusions is an important issue in the management of malignancy patients. Generally, cytologic examination is a routine diagnostic technique that provides cost-effective means with almost no side effects for the patients 2. However, morphological interpretation of cytology for malignancy diagnosis is sometimes inconclusive. Ancillary techniques are often needed for accurate diagnosis of malignant cells 3–9. Thus, it is important to develop a simple method which can warrant high accuracy for malignancy detection.

Recently, it has become of interest in developing fluorescence probes, which can selectively illuminate malignant cells 10–16. Among them, a small organic compound, 3,6-Bis(1-methyl-4-vinylpyridinium)carbazole diiodide (BMVC), shows a strong fluorescence contrast between cancer cells and normal cells due to two distinct properties: a significant increase of the fluorescence quantum yield upon interaction with DNA 17 and a dramatic difference in the intracellular localization between cancer cells and normal cells 18,19. In normal cells, BMVC is retained in the lysosomes after endocytosis, whereas in cancer cells, BMVC escapes from lysosomes and localizes to the nucleus or the mitochondria, where it binds to DNA and shows enhanced fluorescence. These properties illustrate the potential role of fluorescence probe for malignancy diagnosis.

In this study, we developed a cell-collecting kit for the use of BMVC staining and combined ImageJ program to digitize BMVC fluorescence intensity, hereafter named BMVC test. Using BMVC test for the detection of malignant pleural effusions, we overcame the limitations of bare BMVC staining in clinical test and reduced the ambiguity in morphological interpretation of cytologic smear due to intra-observer or inter-observer variability.

In general, autofluorescence of red blood cells (RBCs) and some contaminants or BMVC fluorescence upon interaction with contaminants in the pleural effusion may cause false positive results. Moreover, dead cells can contribute false positive signals severely because BMVC can enter the nucleus of dead cells and show strong fluorescence 19. On the other hand, insufficient viable cells may lead to false negative and non-diagnostic results. Accordingly, a pre-processing of sample for collecting living cells from pleural effusion is a prerequisite to BMVC test. Here a simple cell-collecting kit based on size filtration and surface selection is designed for cell collection by packing surface modified silica gel particles into a pipet tip as a column. Components in the samples with sizes near to the dimensions of pores and tunnels formed by silica gels are likely to accumulate in the column, while impurities which are much smaller or larger can be excluded. In addition, for enriching cell populations, the positive charge modification on the silica gel particles facilitates living cell adhesion and decreases the loss of living cells due to the flush of sample fluid, while dead cells without membrane charge could be washed away from the column. The surface modification can also inhibit the non-specific adhesion between silica gel surface and BMVC molecule due to charge repulsion, thus increase signal-to-noise ratio.

The cytologically diagnostic criteria of malignant pleural effusion include large cell clusters, unusual cell shape, secretory cytoplasmic vacuoles/cytoplasmic keratinization, large cells with high nuclear to cytoplasmic ratios (N/C ratios), irregular nuclear contour, hyperchromasia, coarse chromatin and prominent nucleolus. Obviously, the diagnostic yield depends upon the experiences and skills of the cytologists 20. Sometimes, ambiguities in interpretation of the body fluid cytology for the malignancy diagnosis cannot be avoided in cytologic examination. Cytologic results such as “atypical”, “indeterminate”, and “suspicious for malignant cells” provide limited information to clinicians. Ancillary tests including flow cytometry with DNA analysis 21–23 or immunocytometry 24, electron microscopic examination 25,26, and immunohistochemical studies 27–29 may be used. However, there are still some imperfections such as complicated and long procedures or limitation to certain types of malignancy. BMVC test is a fast and objective method, where the fluorescence signal is digitized by program and the digital number gives a clear-cut result without any grey zone. Also it is not limited to a certain cancer type 18.

In order to apply BMVC test to the diagnosis of malignant pleural effusions and serve as a credible reference for clinicians, we conducted this study to examine the accuracy of the BMVC test in detection of malignant pleural effusions.

## Materials, Patients, and Methods

### Kits and device

To coat poly-l-lysine on the surface of silica gel particles, 0.5 g silica gel particles (60-200 *μ*m, Sigma-Aldrich, St. Louis, MO) were added into 3 mL poly-l-lysine solution (0.1 mg/mL, Sigma-Aldrich) at 37°C for 24 h. We then added 20 *μ*L of the silica gel solution into a pipet tip (volume 0.1-10 *μ*L, Labcon, San Francisco, CA). Silica gel particles subsided immediately and formed a 0.5 cm column. After packing the small pipet, the samples with the amount of 200 *μ*L were first stained by 20 *μ*mol/L BMVC solution for 3 min and were then loaded in the pipet tip. Pressure was applied to allow the samples to flow through the silica gel column. Due to the size filtration of tunnels and surface selection of poly-l-lysine coating, living cells were collected in the column. Simultaneously, a control sample containing only 20 *μ*mol/L BMVC was flowed through another column.

**Figure 1:**
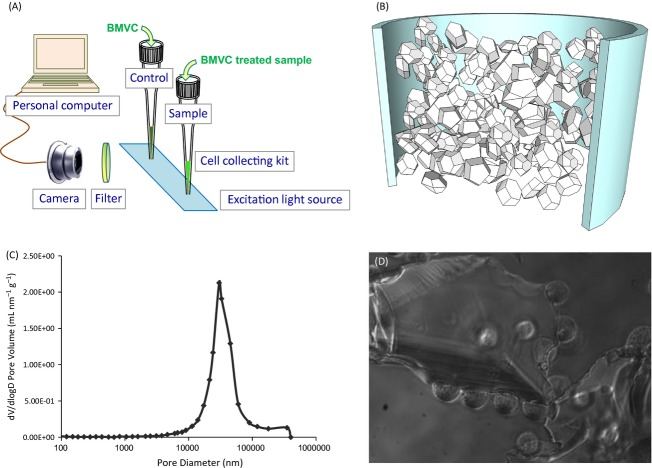
(A) Concept of BMVC test. (B) A pipet tip packed with 60-200μm silica gel particles used as a cell kit. Sample fluid flows through the column by the pressurization. (C)The pore size distribution between silica gel particles. Mercury porosimetry was used to determine the pore size, which is distributed between 17 and 60 μm. (D) CL1-0 cells attached onto poly-L-lysine coated silica gel particles. The transparent polyhedron with large size is the silica gel particle, where the spheres with small size adsorbed on the polyhedron are CL1-0 cells.

As shown in Figure 1A, the loaded tip was inserted into a black box and irradiated by a 450 nm light source, which is an ultraviolet lamp (Spectroline, Westbury, NY) included at the bottom of the box. The fluorescence contrast was achieved where target cells emitted strong fluorescence while non-specific cells did not. A digital camera (Canon G11, Tokyo, Japan) together with a 535DF35 filter (Omega Optical, Brattleboro, VT) was used to record the fluorescence images (5 sec shutter speed, F2.8 aperture). Recorded images were analyzed by ImageJ program (Wayne Rasband, National Institute of Health, Bethesda, MD). The images were transferred to RGB stack type, and only green colored pixels resulting from BMVC were counted and averaged to calculate the intensity of the columns. The final results were obtained by subtracting the intensities of control columns from that of sample columns.

### Mercury Porosimeter

Autopore 9520 Mercury Porosimeter (Micromeritics, Norcross, GA) is an analytical technique used to determine pore size distribution 30.

### Cell lines

CL1-0, human lung adenocarcinoma cell, was isolated by Professor Pan-Chyr Yang 31 and was kindly provided by Professor Chin-Tin Chen at National Taiwan University. MRC-5, human normal lung fibroblast, were purchased from ATCC (American Type Culture Collection). CL1-0 were grown in RPMI-1640 medium supplied with 10% FBS. MRC-5 were grown in MEM medium supplied with 10% FBS. All cells were cultured at 37°C in 5% CO_2_.

### Dead cell preparation

To examine the performance of BMVC in dead normal cells, MRC-5 was fixed before BMVC test 32. Cells were washed by cold PBS twice then fixed by Methonal/Aceton (1:1) mixture at −20°C for 5–10 min. Dead cells were confirmed by Trypan Blue under microscopy.

### Patient population

Eighty-one patients with pleural effusions were recruited prospectively from the National Taiwan University Hospital between June 2012 and December 2012. A total of 110 pleural effusions were collected. The clinical presentation, laboratory data, radiological findings, and histopathologic reports were reviewed. This study protocol was approved by the Institutional Review Board at the National Taiwan University Hospital (No. 201003082R) and informed consent was obtained from the patients.

### Patient sample preparation, cytologic examination and BMVC test

In general, 10 mL pleural effusion was collected from a patient by thoracentasis and centrifuged for 5 min (250 × *g*). Subsequently, the buffy coat was withdrawn by droppers. One drop was used for double-blind cytologic examination according to routine cytology laboratory methods, and Riu's stain (a modified Romanowsky stain, Muto Pure Chemicals, Tokyo, Japan) was performed on all cytologic smears 33. The remaining cell pellet in the dropper was dispersed in 200 *μ*L PBS buffer for BMVC test.

### Statistical analysis

The sensitivity, specificity, positive and negative predictive values of BMVC test in detection of malignant pleural effusions was calculated using standard statistical methods.

## Results

### Cell kit

A simple cell kit based on size filtration and surface selection is designed for improving the accuracy of BMVC staining in detection of malignant pleural effusion. To exclude impurities by size filtration in the cell kit, 60-200 *μ*m silica gel particles with surface coating were loaded into a pipet tip and shaped into a column structure, as shown in Figure 1B. The pore size distribution measured by mercury porosimetry is around 17 to 60 *μ*m, as shown in Figure 1C. Impurities with small size, such as RBCs, cell debris, and bacteria, are washed away, while impurities with large size, such as contaminants and tissue, are excluded since they cannot enter the pores and tunnels of the column.

The purpose of surface coating of silica gel particles is to utilize surface selection for increasing living cell collection. For simplicity and low cost, the coating material chosen is poly-l-lysine. Figure 1D shows the cells adsorbed on silica gel particles after injection of the CL1-0 cells dispersed in RPMI-1640 (10% FBS) medium through the silica gel column. It clearly shows that cells adhered to the particles or gathered around the particles, implying that poly-l-lysine coated silica gel particles could help collect living cells.

### BMVC test in cell lines

**Figure 2:**
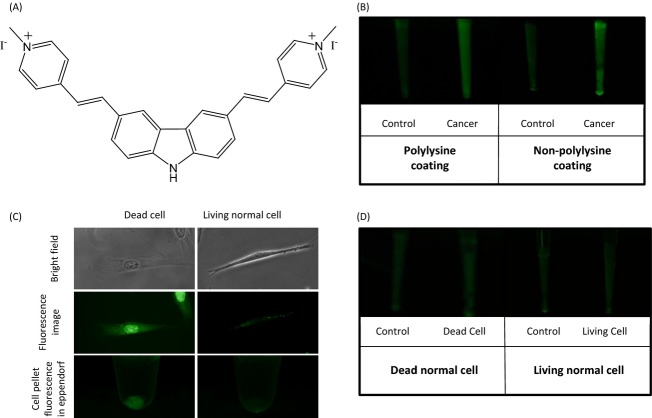
(A) The chemical structure of BMVC molecule. (B) The control intensities and results after subtracting control intensities of poly-L-lysine coated gel column and non-coated gel column. The signals from left to right: 21, 59, 26, and 56. (C) Living and dead MRC-5 cells treated by 20 μM BMVC. From top to bottom: bright field cell microscopic images, fluorescent cell microscopic images, fluorescence of cell pellet in eppendorfs. (D) BMVC test results of living and dead MRC-5 cells. The signals are 19, 19·5, 20, and 20.4, from left to right.

The chemical structure of BMVC is shown in Figure 2A. Since BMVC is positively charged, the positive charge of poly-l-lysine provides charge repulsion for BMVC which inhibits the non-specific adhesion between silica gel surface and BMVC molecule. Thus, the poly-l-lysine coating can increase signal-to-noise ratio through increasing the living cell collection and decreasing BMVC nonspecific adhesion. In vitro experiments with or without poly-l-lysine coating as shown in Figure 2B, the control signals are 21 and 26 while the sample signals are 59 and 56, respectively. The higher control signal from experiment without poly-l-lysine coating is due to lack of repulsion between free BMVC molecules and silica gel particles. Also, the results after subtracting control signals from sample signals decreased from 38 to 30 due to lack of attraction between cell membranes and non-coated silica gel particles. These results indicated that the signal-to-noise ratio can be promoted by poly-l-lysine coating.

In addition, BMVC shows strong fluorescence intensity in dead MRC-5 cells even though they are normal cells because BMVC can enter their nuclei and bind with DNA, as shown in Figure 2C. To overcome this limitation of bare BMVC staining in clinical test, it is critical to eliminate dead cells for BMVC test to prevent false positive. While dead cells without membrane charge could easily be washed away from the column, the negative charges of living cell membrane are attracted by the positively charged poly-l-lysine coated silica gel particles 34,35. Although dead normal cells show much stronger fluorescence intensities than living normal cells, our results showed that similar fluorescence signals were detected from the two silica gel columns containing either dead or living cells, as shown in Figure 2D. This suggests that the use of cell kit can eliminate the influence of dead cells by washing away unwanted dead cells and thus reduce the false positive signals.

**Figure 3:**
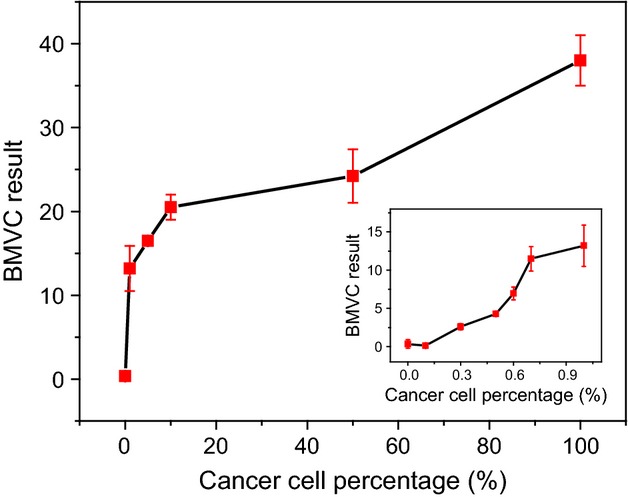
Quantitative measurements of cell ratios of CL1-0 cancer cells within MRC-5 normal cells. The inset shows that cancer cells can be detected as low as 0.3%.

To examine the detection limit of the BMVC test, Figure 3 shows a plot of cell ratios of CL1-0 cancer cells to MRC-5 normal cells versus test value of BMVC test in vitro. The total cell numbers were one million, and the ratios of cancer cells to normal cells increased from 0% to 100%. Our results showed that the BMVC test allows us to detect the cancer cells when the ratio is larger than 1%, and possibly down to 0.3% based on the fluorescence intensity.

### Patient population

In this work, a total of 110 pleural effusions were collected from 81 patients (male: 48, 59.2%). The mean age was 66.4 years old (range: 32-90 years). The underlying diseases of these patients included 69 malignant diseases and 12 benign ones. Note that malignant cells may not invade pleura or exfoliate into pleural cavity 36, thus specimens of pleural effusions sometimes show negative results for cancer patients.

### BMVC test in clinical pleural effusion

**Figure 4:**
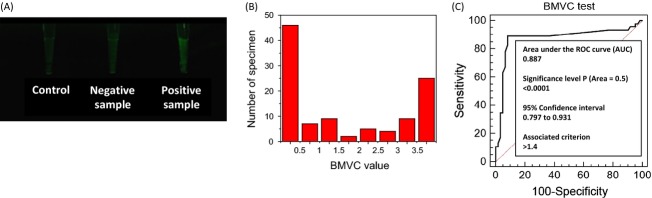
(A) The positive and negative patient samples compared with control column. Positive sample has the strongest fluorescence intensity. (B) The histogram of the BMVC test value from pleural effusion of patients. (C) The receiver operating characteristic (ROC) curve comparing to double-blind cytologic examination.

Among the 110 specimens, two samples were reported as unsatisfactory specimens for BMVC test. Without these 2 specimens, a total of 108 pleural effusions from 80 patients were analyzed in this study, who are numbered in Table 1. Among these 80 patients, 63 patients provided single specimen and 17 patients provided multiple specimens collected on different days. All the results from BMVC test together with those from cytologic examination are also listed in Table 1. Considering the results from cytologic examination as gold standard, there were 47 positive, 60 negative, and 1 suspicious specimen. Without considering the suspicious specimen (patient number 22), 9 out of 107 specimens showed inconsistent results between BMVC test and cytologic examination, which were 4 false positive specimens and 5 false negative specimens. Figure 4A shows fluorescence images of a positive sample and a negative sample together with a control sample. The fluorescence intensity was analyzed by ImageJ program. The difference of fluorescence intensity between sample and control is 15.0 for the positive sample and 1.1 for the negative sample. Figure 4B and C show a histogram of BMVC test value from pleural effusion of patients and the receiver operating characteristic (ROC) curve, respectively. Comparing to double-blind cytologic examination, the ROC curve indicates that this method should have an impressive predictive value. Thus, a threshold value of 1.4 was applied to differentiate malignant and benign specimens based on distribution of BMVC test results and ROC curve analysis. The area under ROC curve presents the sensitivity and the specificity of this method 37, and the value of 0.88 indicates that this method provides a high accuracy for detection of malignant pleural effusions. Also, the significance level (*P*) is under 0.0001, which suggests that this method is a reliable tool for malignancy detection.

**Table 1:** The BMVC test results. The table includes the patient number, number of specimen collected, and the comparison of BMVC test with cytologic examination.

Patient number	Number of specimen collected	Results of BMVC test and cytologic examinaiton^1^	Number of consistent result	Number of inconsistent result^2^	Clinical diagnosis	Histopathologic results of pleural biopsy	Results of cell block study of pleural effusion
1	4	(+,+) (+,+) (−,+) (−,−)	3	1 (FN)	Lung adenocarcinoma	None	Adenocarcinoma, metastatic
2	4	(+,−) (−,−) (−,−) (−,−)	3	1 (FP)	Lung squamous carcinoma	None	None
3	3	(+,+) (+,+) (+,+)	3	0	Lung adenocarcinoma	None	Adenocarcinoma, metastatic
4	3	(+,+) (+,+) (+,+)	3	0	Lung adenocarcinoma	None	None
5	3	(−,−) (+,+) (+,+)	3	0	Lung adenocarcinoma	None	Adenocarcinoma, metastatic
6	3	(−,−) (−,−) (−,−)	3	0	Multiple myeloma with cardiac amyloidosis	None	None
7	3	(−,−) (−,−) (−,−)	3	0	Lung sarcomatoid carcinoma	None	None
8	3	(−,−) (−,−) (−,−)	3	0	Bilateral massive pleural effusion, nature undetermined	Chronic inflammation	None
9	3	(+,+) (−,+) (+,+)	2	1 (FN)	Lung adenocarcinoma	None	Adenocarcinoma, metastatic
10	2	(+,+) (+,+)	2	0	Breast, ductal carcinoma	None	None
11	2	(+,+) (+,+)	2	0	Breast, invasive carcinoma	None	None
12	2	(+,+) (−,−)	2	0	Breast, ductal carcinoma	Carcinoma, metastatic	None
13	2	(−,−) (−,−)	2	0	Coronary artery disease with congestive heart failure	Chronic pleuritis	None
14	2	(−,−) (−,−)	2	0	Lymphoma	No evidence of malignancy	Rare atypical lymphoid cells
15	2	(−,−) (−,−)	2	0	Lung adenocarcinoma	Chronic fibrosing pleurisy with atypical cells	None
16	2	(+,+) (−,+)	1	1 (FN)	Lung adenocarcinoma	None	Adenocarcinoma, metastatic
17	2	(−,−) (−,+)	1	1 (FN)	Lung adenocarcinoma	None	None
18	1	(+,−)	0	1 (FP)	Uterus, cervix, squamous carcinoma	None	None
19	1	(+,−)	0	1 (FP)	Sigmoid colon cancer	None	None
20	1	(+,−)	0	1 (FP)	Lung, squamus cell carcinoma	None	None
21	1	(−,+)	0	1 (FN)	Lung adenocarcinoma	None	None
22	1	(+,±)	0	1	Rectal cancer	Adenocarcinoma, metastatic	None
23	1	(+,+)	1	0	Breast, ductal carcinoma;P:20121106	Adenocarcinoma, metastatic	None
24	1	(+,+)	1	0	Melanoma	Malignant melanoma, metastatic	
25	1	(+,+)	1	0	Lung adenocarcinoma	None	None
26	1	(+,+)	1	0	Breast, ductal carcinoma	None	None
27	1	(+,+)	1	0	Lung adenocarcinoma		Adenocarcinoma, metastatic
28	1	(+,+)	1	0	Ovarian serous carcinoma	None	None
29	1	(+,+)	1	0	Lung adenocarcinoma	None	Adenocarcinoma, metastatic
30	1	(+,+)	1	0	Lung adenocarcinoma	None	Adenocarcinoma, metastatic
31	1	(+,+)	1	0	Lung adenocarcinoma	None	Adenocarcinoma, metastatic
32	1	(+,+)	1	0	Lung adenocarcinoma	None	None
33	1	(+,+)	1	0	Lung adenocarcinoma	None	Adenocarcinoma, metastatic
34	1	(+,+)	1	0	Lung adenocarcinoma and pleomorphic carcinoma	None	Adenocarcinoma, metastatic
35	1	(+,+)	1	0	Lung squamous cell carcinoma	None	None
36	1	(+,+)	1	0	Lung adenocarcinoma	None	None
37	1	(+,+)	1	0	Lung adenocarcinoma	None	Adenocarcinoma, metastatic
38	1	(+,+)	1	0	Lung adenocarcinoma	None	Adenocarcinoma, metastatic
39	1	(+,+)	1	0	Lung adenocarcinoma	None	None
40	1	(+,+)	1	0	Thyroid papillary carcinoma	None	None
41	1	(+,+)	1	0	Lung adenocarcinoma	None	Adenocarcinoma, metastatic
42	1	(+,+)	1	0	Lung adenocarcinoma	None	Adenocarcinoma, metastatic
43	1	(+,+)	1	0	Lung adenocarcinoma	None	None
44	1	(+,+)	1	0	Lung adenocarcinoma	None	Adeno
45	1	(+,+)	1	0	Lung adenocarcinoma	None	None
46	1	(+,+)	1	0	Lung adenocarcinoma	None	Adenocarcinoma, metastatic
47	1	(−,−)	1	0	Lung, small cell carcinoma	None	None
48	1	(−,−)	1	0	Pancreatic head cancer	None	None
49	1	(−,−)	1	0	Lung adenocarcinoma	None	None
50	1	(−,−)	1	0	Lung adenocarcinoma	None	None
51	1	(−,−)	1	0	Lung lymphoepithelioma-like carcinoma	None	None
52	1	(−,−)	1	0	Tuberculous pleurisy	None	None
53	1	(−,−)	1	0	Lung lymphoepithelioma-like carcinoma	None	None
54	1	(−,−)	1	0	Lung adenocarcinoma	None	None
55	1	(−,−)	1	0	Anterior mediastinum, invasive thymoma	None	None
56	1	(−,−)	1	0	Pneumonia	None	None
57	1	(−,−)	1	0	Chronic myeloid leukemia	None	None
58	1	(−,−)	1	0	Lung adenocarcinoma	None	None
59	1	(−,−)	1	0	Chronic inflammation	None	None
60	1	(−,−)	1	0	Malnutrition	None	None
61	1	(−,−)	1	0	Lung squamous cell carcinoma	None	None
62	1	(−,−)	1	0	Anterior mediastinum, invasive thymoma	None	None
63	1	(−,−)	1	0	Lung adenosquamous carcinoma	None	None
64	1	(−,−)	1	0	Esophageal squamous cell carcinoma	None	None
65	1	(−,−)	1	0	Right subphrenic abscess with reactive pleural effusion	None	None
66	1	(−,−)	1	0	Lung adenocarcinoma	None	None
67	1	(−,−)	1	0	Lung squamous cell carcinoma	None	None
68	1	(−,−)	1	0	Lung adenocarcinoma	None	None
69	1	(−,−)	1	0	Lung adenocarcinoma	None	None
70	1	(−,−)	1	0	Esophagus and larynx, squamous cell carcinoma	None	None
71	1	(−,−)	1	0	Lung squamous cell carcinoma	No evidence of malignancy	None
72	1	(−,−)	1	0	Aortic valve regurgitation with congestive heart failure	None	None
73	1	(−,−)	1	0	Lung adenocarcinoma	None	None
74	1	(−,−)	1	0	Chronic inflammation	Chronic inflammation	None
75	1	(−,−)	1	0	Esophageal squamous cell carcinoma	None	None
76	1	(−,−)	1	0	Lung adenocarcinoma	None	None
77	1	(−,−)	1	0	Left hydropneumothorax, etiology to be determined	Chronic inflammation	None
78	1	(−,−)	1	0	Pulmonary edema and bilateral pleural effusion, suspected heart failure and chronic kidney disease related	None	None
79	1	(−,−)	1	0	Chylothorax	None	None
80	1	(−,−)	1	0	Tuberculous of lung	None	None

1 The multiple parentheses are test results performed on different days for each patient; the left sign in the parenthesis refers to the result of BMVC test, while the right one refers to result of cytologic examination; + refers to positive for malignant cells, − refers to negative for malignant cells, and ± refers to suspicious for malignant cells.

2 FN refers to false negative result of BMVC test by considering cytologic examination as gold standard, and FP refers to false positive result of the BMVC test.

Table 2 shows the diagnostic results of BMVC test compared with cytologic examination. The BMVC test achieved a 91.6% (98/107) diagnostic accuracy with a sensitivity of 89.4% (42/47) and a specificity of 93.3% (56/60). In addition, the positive predict value is 91.3% (42/46) and the negative predict value is 91.8% (56/61).

**Table 2:** Diagnostic yield of BMVC test compared with cytologic examination

	BMVC test		
	Positive	Negative	Total
Cytologic examination			
Positive	42	5	47
Negative	4	56	60
Total	46	61	107

## Discussion

### Non-diagnostic specimens

Two out of 110 specimens were reported as unsatisfactory since the BMVC values were more negative than −9 after subtracting control signals. Both of these two specimens showed dark-chocolate color due to the large amount of old blood contained in the specimen, thus the fluorescence of BMVC is significantly interfered by the deep color.

### Reproducibility

As shown in Table 1, among the 17 patients (patient number 1–17) who provided multiple specimens (total of 45 specimens), the results of BMVC test were consistent with cytologic examination for 12 patients, who provided total of 30 specimens. Patient number 3, 4, 10, and 11 provided a total of 10 specimens, which were all positive results for both BMVC test and cytologic examination. Also, there were a total of 15 specimens, which were all negative results for both BMVC test and cytologic examination, provided by patient number 6, 7, 8, 13, 14, and 15. Even though the specimens were collected on different days from the same patients, the results of BMVC test were stable and consistent with cytologic examination.

For patient number 5 who provided three specimens, there were no malignant cells in cytologic examination in the first specimen. However, malignant cells appeared in the second and third specimens. The results of BMVC test also changed in these three specimens and stayed consistent with cytologic examination. Similarly, for patient number 12 who provided 2 specimens, both results from BMVC test and cytologic examination changed from positive to negative. Therefore, BMVC test shows the reproducibility and reliability in comparison with cytologic examination.

### False negative specimens

Among the 107 effective specimens, there were five specimens reported as false negative results, which were from patient number 1, 9, 16, 17, and 21.

The most likely reason of the discrepancy for specimens from patient number 9, 16, and 17 is due to specimen sampling error. It is known that the malignant cells do not distribute homogeneously in pleural effusions, especially when malignant cells do not exfoliate large amount of cells from the pleura into the pleural effusions. In these cases, the pleural effusions from these three patients showed very few malignant cells in the smear of cytologic examination. It is likely that the specimen for BMVC test may not contain detectable malignant cells. Thus, the lack of sufficient malignant cells in pleural effusion is the major cause that leads to inaccuracy of the BMVC test.

For the false negative results from patient number 1 and 21, the cell morphology in cytologic examination suggested that there are very few clustered malignant cells. This means that they may be more difficult to be collected in the pores and tunnels of the cell-collecting column. On the other hand, the broad size distribution (17 to 60 *μ*m) of the pores and tunnels facilitate the collection of cells or cell clusters in different sizes; however, sometimes inevitable uneven distribution may lead to insufficient small pores in some columns. When these columns which are not suitable for collecting smaller cell clusters used to test the specimens such as that from patient number 1 and 21, the unsuccessful cell collection may bring about false negative results.

To eliminate these two types of probable error, we can use more than one silica gel columns to deal with more pleural effusion samples because of the high throughput of the BMVC test. More cell samples can be acquired by silica gel columns and reduce false negative errors from a single patient by avoiding inhomogeneous specimen.

### False positive specimens

Among the 107 effective specimens, there were four specimens reported as false positive results, which were provided by patient number 2, 18, 19, and 20.

One possibility is that BMVC compound may emit non-specific fluorescence when being trapped in highly sticky fluid since increasing the viscosity of local environments could suppress nonradiative transitions of the torsional motion at the vinyl group of BMVC 38. For the first specimen from patient number 2 and the specimens from patient number 19 and 20, the false positive results may be due to the nature of highly sticky pleural effusions from these three patients. For example, patient number 20 were diagnosed as empyema. The sticky condition caused by components in empyema fluid such as fibrin may generate non-specific fluorescence of BMVC; besides, the highly sticky fluid may slow down the flow of specimen or even block the tunnels in cell collecting columns. Since empyema fluid contains many pus cells, dead cells, cell debris, and bacteria that may also be illuminated by BMVC, these unwanted components may not be washed out from the column due to the viscous sample, thus generate false positive signals.

Although some specimens were reported as false positives, the BMVC test results are not necessarily wrong. The result of specimen from patient number 18 for which the BMVC test value was 3, indicating that there may be metastatic malignant cells in pleural effusion. Even though the cytologic examination test did not observe any malignant cells, the clinical diagnosis is cervical cancer with abdominal lymphadenopathy and multiple metastases at pleural and peritoneal region. In this case, BMVC test may not be wrong even though the results are inconsistent with cytologic examination.

### Potential of serving as a credible reference

In some cases, BMVC test showed the potential to provide an objective and clear-cut reference without grey zone for cytologists and clinicians.

Since malignant cells may not invade pleura, specimens of pleural effusions sometimes show negative results for cancer patients. Patient number 7 is a case of lung cancer, diagnosed as stage Ia sarcomatoid carcinoma. This patient has received adjuvant chemotherapy and radiotherapy after video-assisted thoracoscopic surgery (VATS) lobectomy. He was first recruited last July and found a sheet of small cuboid cells with high N/C ratio on the cytologic smear of the first pleural effusion, which was considered atypical in comparison with other cells on the smear. Although the cytologist reported this specimen as a negative result, the description such as “a sheet of small cuboid cells with high N/C ratio was found” is not clear for clinicians. Meanwhile, BMVC test provided a clear-cut value of 1, which means this specimen contained no malignant cells. This patient has been followed up since recruitment. Both the BMVC test and cytologic examination of next 2 pleural effusions showed negative results meaning there were no malignant cells in this patient's pleural effusion even though he is a cancer patient. In this case, BMVC test may provide another reliable reference for clinicians, or help cytologists make a more accurate report during the review of cytologic morphology.

Additionally, for the specimen from patient number 22, the double-blind cytologic examination showed a few cells with cellular enlargement, eccentric nucleus and transparent cytoplasm. It was difficult to determine if these signet ring form cells were malignant cells or not, especially when there were very few abnormal cells on the cytologic smear. Thus, the cytologic examination of this specimen was reported as “suspicious”, where clinical diagnosis of this patient is rectal cancer with liver, lung, pleural, thymus and brain metastases. This suggested the limitation of cytologic interpretation. In this case, the BMVC test gave a value of 6 indicating the presence of malignant cells. Since BMVC test is sensitive, digitalized and clear-cut, no ambiguous results were produced. With the support of BMVC test, clinicians can acquire more accurate information from the pleural effusion even though the case was reported as undetermined in cytologic examination.

### Integration of BMVC test into clinical practice

To integrate BMVC test into clinical practice for the evaluation of pleural effusions, there is a simple procedure that can be coupled to the routine process. After pleural effusion is collected by thoracentasis and centrifuged, the buffy coat is withdrawn by a dropper and one drop is used for routine cytologic smear test. Meanwhile, the remaining cell pellet in the dropper needs be dispersed in PBS buffer and stained by BMVC solution for 3 min. After flowing through the cell-collecting kit, the sample kit together with control kit in a black box is irradiated by a light source. A digital camera with filter is used to record the images which will be analyzed by ImageJ program immediately. The instant information can be provided for clinicians in out-patient department, or for cytologists during review of smears.

In summary, the 89.4% sensitivity and the 93.3% specificity in this study undoubtedly indicate that the BMVC test provides a reliable tool for detection of malignant pleural effusions. In addition, the quantitative and objective test result is immediately available to the clinician right after the test has been examined. We believe that this simple, fast, and objective technique with high accuracy at low cost can play an important role in malignancy diagnosis and management. Since the pleural effusions may recur and have to be drained out for patients, the BMVC test is particularly useful for the follow-up examination.
